# Inguinodynia following inguinal hernia repair: a comparative analysis of open Lichtenstein and laparoscopic TAPP techniques in a two-center cohort

**DOI:** 10.3389/fsurg.2025.1590855

**Published:** 2025-05-22

**Authors:** Anas Aljaiuossi, Saleh A. Ba-shammakh, Mohammad Bani Hani, Musab Al-A'athal, Mohammad Abu-Hussein, Mohammed Sobh, Mohammad Yousef, Zaid Mesmar, Raad Dowais, Aseel W. Ababneh, Ahmad Kenani, Mohammad Ababneh, Sameh Alawneh, Rawa'a Al-Ibrahim, Saja Al-Shdooh, Thikrayat Daoud

**Affiliations:** ^1^ Department of General Surgery and Anesthesia, Faculty of Medicine, Yarmouk University, Irbid, Jordan; ^2^ Department of General Surgery, Ministry of Health, Amman, Jordan; ^3^ Department of General Surgery and Urology, Jordan University of Science and Technology, Al Ramtha, Jordan; ^4^ Department of Public Health and Community Medicine, Jordan University of Science and Technology, Irbid, Jordan; ^5^ Faculty of Medicine, Jordan University of Science and Technology, Irbid, Jordan; ^6^ Department of Family Medicine, Jordan University of Science and Technology, Irbid, Jordan

**Keywords:** inguinal hernia, inguinodynia, laparoscopic repair, Lichtenstein hernioplasty, TAPP, chronic pain

## Abstract

**Background:**

Chronic postoperative groin pain (inguinodynia) is a major determinant of patient-reported outcomes after inguinal hernia repair. Evidence comparing open Lichtenstein hernioplasty (LICH) with laparoscopic trans-abdominal pre-peritoneal repair (TAPP) in everyday practice remains limited.

**Methods:**

We performed a retrospective, two-centre cohort study of adults who underwent elective primary or recurrent inguinal hernia repair at King Abdullah University Hospital and Princess Basma Hospital (2010–2020). Patients were assigned to LICH (*n* = 107) or TAPP (*n* = 103). The primary endpoint was inguinodynia, defined as pain persisting ≥3 months post-operatively. Secondary endpoints included length of stay (LOS) and surgical complications. Multivariate logistic regression adjusted for age, body-mass index, smoking, hypertension, diabetes and benign prostatic hyperplasia.

**Results:**

Baseline demographics were comparable, although hypertension (28% vs. 15.5%, *p* = 0.045) and current smoking (41.1% vs. 25.2%, *p* = 0.020) were more prevalent in the LICH group. Inguinodynia occurred in 23 LICH patients (21.5%) and 9 TAPP patients (8.7%) (*p* = 0.013). After risk adjustment, LICH remained an independent predictor of chronic pain (adjusted OR = 2.98; 95% CI 1.28–6.97; *p* = 0.012). Median LOS was slightly longer after LICH (2.40 ± 1.40 days) than TAPP (2.02 ± 0.89 days; *p* = 0.039). Overall rates of hematoma, seroma, surgical-site infection and early recurrence were low and did not differ significantly between techniques.

**Conclusion:**

TAPP markedly lowers chronic pain and shortens hospital stay without extra morbidity, supporting its preferential use when expertise permits.

## 1 Introduction

Inguinal hernia is a common disease that affects nearly 25% of men and less than 2% of women over their lifetime and the counter is still counting (1). Around 800,000 inguinal hernia repairs are made annually in the United States, out of the more than 20 million that are done worldwide (2). Inguinal hernias are classified as either direct or indirect based on whether the hernia sac bulges through the inguinal canal's posterior wall directly (direct hernia) or passes through the internal inguinal ring alongside the spermatic cord, following the coursing of the inguinal canal (indirect hernia) (3).

The latest International Guidelines of the Hernia-Surge Group recommend the totally extraperitoneal patch plasty (TEP), transabdominal preperitoneal patch plasty (TAPP), and Lichtenstein techniques out of a total of more than 100 different repair techniques for inguinal and femoral hernias, which are classified as tissue repair, open mesh repair, and laparo-endoscopic mesh repair (4, 5). Each procedure differs from the other by several factors For example the advantages of the Liechtenstein approach include its low cost, low operating time, and ease of learning (6).

One of the most important complications in the postoperative period of inguinal hernioplasties is inguinodynia, an ongoing, moderate-to-severe groin pain that has persisted for longer than three months after an inguinal hernia repair, the prevalence varies among studies its prevalence can be as high as 62.9% (7, 8). According to their relationship to nerve injury and the mesh, the pain is categorized as neuropathic and non-neuropathic, respectively (9). Management of Inguinodynia constitutes challenging issues for the clinician as it has an impact on the health system and economy according to the Patient Reported Outcome Measurement which has underlined the importance of chronic postoperative inguinal pain, and its great limitations in everyday life (10).

Studies suggest that inguinodynia rates are typically lower after minimally invasive laparoscopic hernia repair TAPP in contrast to open surgery LICH (11). After laparoscopic repair, inguinodynia tends to be milder and shorter duration compared to open surgery. Symptoms may manifest as mild discomfort or occasional twinges in the groin area, resolving more rapidly due to the less invasive nature of the procedure (12, 13). A review of the most important research indicates that individuals who undergo laparoscopic hernia repair typically achieve a faster resumption of daily activities and work, experiencing less interference with their daily routines compared to those undergoing open surgery (14). This can be attributed to decreased postoperative pain and earlier alleviation of inguinodynia symptoms. Our study will provide evidence-based insights that can guide surgical method selection to minimize the risk of chronic postoperative pain.

The lack of sufficient papers and studies, particularly in evaluating the risk of inguinodynia between open and minimally invasive inguinal hernia repair, specifically focusing on Lichtenstein hernioplasty (LICH) and laparoscopic transabdominal preperitoneal repair (TAPP), highlights a substantial gap in current research. This gap underscores the need for further research and investigation into this important aspect of hernia repair methodologies.

This research holds significance in providing essential insights and direction for surgical practices tailored to individual patient needs. Additionally, it emphasizes the importance of specific patient care approaches, underscoring the elective nature of the surgeries under scrutiny to mitigate potential biases. This underlines the critical role of informed decision-making in optimizing patient outcomes and ensuring quality care delivery.

## 2 Methods

### 2.1 Study design and setting

This study employed a retrospective cohort design, conducted across two medical centers in Jordan: King Abdullah University Hospital (KAUH) and Princess Basma Hospital (PBH). Ethical approval was granted by the Institutional Review Board (IRB) of Yarmouk University (Reference No. DSR/2024/024). All patient data were anonymized to protect confidentiality, and a Certificate of Confidentiality was not required due to the study's retrospective and anonymized nature. Written informed consent for the use of anonymized patient data for research purposes was routinely obtained from all patients upon admission.

### 2.2 Study population

Patients undergoing elective inguinal hernia repair at the two centers between January 2010 and December 2020 were evaluated for inclusion. Eligible participants included adults aged 18–70 years who had undergone either laparoscopic transabdominal preperitoneal (TAPP) repair or open Lichtenstein tension-free hernioplasty (LICH). Additionally, a minimum postoperative follow-up period of two years was required. Exclusion criteria included emergency hernia repairs and patients with significant pre-existing neuropathies or neurological deficits.

### 2.3 Data collection

Two independent reviewers—one expert in laparoscopic surgery and another in open hernia repair—performed data extraction from electronic medical records. Demographic variables collected included age, gender, and body mass index (BMI). Clinical comorbidities such as diabetes mellitus, hypertension, asthma, chronic obstructive pulmonary disease (COPD), and benign prostatic hyperplasia (BPH) were documented. Social history, specifically smoking status and alcohol consumption, was also recorded. Hernia-specific details included type, anatomical side, and laterality. Surgical information recorded included technique used (TAPP or LICH), intraoperative complications, postoperative complications, length of hospital stay, and follow-up duration. The primary outcome measure was the incidence and timing of inguinodynia.

Follow-up intervals included routine postoperative assessments at 3, 6, and 12 months, with additional follow-ups extending beyond 18 months for selected patients who required extended monitoring. We retrospectively assessed whether postoperative pain had persisted continuously since the immediate postoperative period. If patients reported no persistent pain at the 3-month follow-up, they were subsequently contacted to confirm whether inguinodynia developed later. Only persistent pain lasting beyond three months was classified as inguinodynia, aligning with standard clinical definitions.

### 2.4 Surgical techniques

The laparoscopic TAPP repair involved general anesthesia, using small abdominal incisions to insert a laparoscope and surgical instruments. After carbon dioxide insufflation, the preperitoneal space was dissected, and a lightweight polypropylene mesh was positioned over the hernia defect. Mesh fixation was achieved using non-absorbable tackers (3–4 tackers per case), supplemented by peritoneal closure with absorbable vicryl sutures. The peritoneum was subsequently closed over the mesh, and the incisions were sutured.

The open Lichtenstein repair (LICH) was performed under either local or general anesthesia. A single incision was made over the hernia site, followed by repositioning or removal of the hernia sac. A lightweight polypropylene mesh was then placed directly over the hernia defect and secured to surrounding tissues without tension, followed by standard closure of the incision.

### 2.5 Definition of inguinodynia

Inguinodynia was clinically defined as chronic postoperative groin pain persisting longer than three months, in line with previously published literature (7). Pain reported within two weeks postoperatively as transient tingling was not classified as inguinodynia. Only persistent pain lasting beyond three months was recorded as inguinodynia. These data were collected through detailed chart reviews, noting reports from routine follow-up visits or patient referrals for persistent postoperative discomfort.

### 2.6 Statistical analysis

Continuous variables were presented as means ± standard deviations (SD), and categorical variables were reported as frequencies and percentages. Normality assumptions were evaluated using the Shapiro–Wilk test. Due to non-normal distribution, continuous variables were compared between groups using the Wilcoxon rank-sum test. Categorical variables were compared using Pearson's chi-square or Fisher's exact test, as appropriate. Welch's *t*-test was used to compare timing of inguinodynia detection and follow-up durations between surgical groups due to confirmed unequal variances by Levene's test. Multivariate logistic regression was performed to adjust for potential confounding variables, including diabetes, hypertension, smoking history, and BPH, to identify independent predictors of inguinodynia. All statistical tests were two-sided, with significance set at a *p*-value of less than 0.05. Data analyses were conducted using R statistical software, version 4.3.2.

## 3 Results

### 3.1 Patient demographics and clinical characteristics

A total of 210 patients were included in this analysis, comprising 103 patients who underwent laparoscopic transabdominal preperitoneal (TAPP) repair and 107 who underwent open Lichtenstein tension-free hernioplasty (LICH) (Table 1).

**Table 1:** Demographics and clinical characteristics of the patients.

Variable	TAPP	LICH	*P*-value
	(*n* = 103)	(*n* = 107)	
Age (years ± SD)	50.3 ± 14.7	52.7 ± 15.4	0.197
Male [*n* (%)]	94 (91.3)	97 (90.7)	0.65
BMI (kg/m^2^ ± SD)	24.8 ± 6.26	25.4 ± 4.75	0.51
Comorbidities			
Diabetes [*n* (%)]	13 (12.6)	24 (22.4)	0.1
Hypertension [*n* (%)]	16 (15.5)	30 (28)	0.045
Asthma [*n* (%)]	0 (0.0)	4 (3.7)	0.12
COPD [*n* (%)]	0 (0.0)	1 (0.93)	1
BPH [*n* (%)]	8 (7.76)	9 (8.4)	1
Type of hernia			
Direct [*n* (%)]	26 (25.2)	27 (25.2)	1
Indirect [*n* (%)]	29 (28.15)	43 (40.2)	0.11
Pantaloon [*n* (%)]	5 (4.9)	6 (5.6)	1
Incisional [*n* (%)]	1 (0.97)	0 (0.0)	0.48
Femoral [*n* (%)]	0 (0.0)	1 (0.93)	1
Recurrent [*n* (%)]	33 (32)	21 (19.6)	0.04
Side of hernia			
Right [*n* (%)]	42 (40.7)	53 (49.5)	0.27
Left [*n* (%)]	34 (33)	47 (43.9)	0.16
Bilateral [*n* (%)]	27 (26.2)	9 (8.4)	<0.001
Social history			
Smoking history [*n* (%)]	29 (28.15)	45 (42.1)	0.06
Current smoker [*n* (%)]	26 (25.2)	44 (41.1)	0.02
Hospital Stay [days ± SD]	2.02 ± 0.89	2.4 ± 1.40	0.039

TAPP, transabdominal preperitoneal; LICH, Lichtenstein hernioplasty; SD, standard deviation; *n*, number BMI, body mass index; COPD, chronic obstructive pulmonary disease; BPH, benign prostatic hyperplasia.

The mean age was comparable between the TAPP and LICH groups (50.3 ± 14.7 vs. 52.7 ± 15.4 years, respectively; *p* = 0.197). No significant difference was found in terms of gender distribution, with males representing 91.3% [*n* = 94] in TAPP and 90.7% [*n* = 97] in LICH (*p* = 0.65). Similarly, BMI did not differ significantly between groups (24.8 ± 6.26 kg/m^2^ in TAPP vs. 25.4 ± 4.75 kg/m^2^ in LICH; *p* = 0.51).

With regard to comorbidities, hypertension was significantly more prevalent in the LICH group [28.0% (*n* = 30)] compared to the TAPP group [15.5% (*n* = 16); *p* = 0.045]. Diabetes mellitus was more frequent in the LICH group [22.4% (*n* = 24)] than in the TAPP group [12.6% (*n* = 13)], although this did not reach statistical significance (*p* = 0.10). Other comorbidities such as asthma [0% (*n* = 0) vs. 3.7% (*n* = 4); *p* = 0.12], COPD [0% (*n* = 0) vs. 0.93% (*n* = 1); *p* = 1.00], and BPH [7.8% (*n* = 8) vs. 8.4% (*n* = 9); *p* = 1.00] showed low prevalence and were evenly distributed between groups.

There were no statistically significant differences between groups regarding hernia types (direct, indirect, pantaloon, incisional, or femoral). However, recurrent hernias were significantly more often treated using the TAPP approach [32.0% (*n* = 33)] compared to LICH [19.6% (*n* = 21); *p* = 0.04]. This difference reflects surgical selection bias favoring laparoscopic repair for recurrent hernia cases rather than actual recurrence rates.

Bilateral hernias were significantly more common in patients treated with TAPP [26.2% (*n* = 27)] than LICH [8.4% (*n* = 9); *p* < 0.001], whereas right-sided [40.7% (*n* = 42) vs. 49.5% (*n* = 53); *p* = 0.27] and left-sided hernias [33.0% (*n* = 34) vs. 43.9% (*n* = 47); *p* = 0.16] did not differ significantly.

Regarding smoking history, a higher proportion of previous smokers was observed in the LICH group [42.1% (*n* = 45) vs. 28.2% (*n* = 29); *p* = 0.06], though not statistically significant. Current smoking status was significantly more prevalent among LICH patients [41.1% (*n* = 44)] compared to TAPP patients [25.2% (*n* = 26); *p* = 0.02].

Mean hospital stay duration was significantly longer in the LICH group (2.40 ± 1.40 days) compared to the TAPP group (2.02 ± 0.89 days; *p* = 0.039), suggesting a shorter postoperative recovery associated with the minimally invasive procedure.

### 3.2 Postoperative complications

Inguinodynia was significantly more frequent in the LICH group [21.5% (*n* = 23)] compared to the TAPP group [8.7% (*n* = 9); *p* = 0.013], representing the most notable postoperative complication (Table 2).

**Table 2:** Complication rates in TAPP vs. LICH Procedures.

Complications (univariable analysis)	TAPP	LICH	*P*-value
	(*n* = 103)	(*n* = 107)	
Intraoperative complications			
Bleeding [*n* (%)]	1 (0.97)	2 (1.87)	–
Iatrogenic bladder injury [*n* (%)]	1 (0.97)	0 (0.0)	–
Postoperative complications			
Inguinodynia [*n* (%)]	9 (8.7)	23 (21.5)	0.013
Abscess [*n* (%)]	0 (0.0)	1 (0.93)	1
Hematoma [*n* (%)]	3 (2.9)	1 (0.93)	0.62
Hypertrophied scar [*n* (%)]	0 (0.0)	1 (0.93)	1
Numbness [*n* (%)]	1 (0.97)	8 (7.5)	0.065
Recurrence [*n* (%)]	1 (0.97)	1 (0.93)	1
Seroma [*n* (%)]	2 (1.94)	2 (1.87)	1
Surgical site infection [*n* (%)]	1 (0.97)	2 (1.87)	1

TAPP, transabdominal preperitoneal; LICH, Lichtenstein hernioplasty; *n*, number.

Other complications were rare and showed no significant differences between groups. These included hematoma [2.9% (*n* = 3) TAPP vs. 0.93% (*n* = 1) LICH; *p* = 0.62], seroma formation [1.94% (*n* = 2) TAPP vs. 1.87% (*n* = 2) LICH; *p* = 1.00], and surgical site infections [0.97% (*n* = 1) TAPP vs. 1.87% (*n* = 2) LICH; *p* = 1.00]. Postoperative numbness was more common in the LICH group [7.5% (*n* = 8)] than in the TAPP group [0.97% (*n* = 1)], approaching statistical significance (*p* = 0.065). Rare complications such as hypertrophic scar, abscess, and recurrence were similarly uncommon.

Intraoperative complications were minimal, with bleeding occurring in one TAPP case [0.97% (*n* = 1)] and two LICH cases [1.87% (*n* = 2)], and one bladder injury in the TAPP group [0.97% (*n* = 1)].

### 3.3 Timing of inguinodynia detection and follow-up duration

The mean onset of inguinodynia symptoms was significantly earlier in LICH patients (2.91 ± 0.85 months) compared to TAPP patients (6.56 ± 3.43 months; *p* = 0.013, Welch's *t*-test), suggesting differential nerve irritation mechanisms between the techniques.

The mean follow-up period was similar for both groups, with no significant difference identified (10.92 ± 2.91 months TAPP vs. 10.74 ± 3.91 months LICH; *p* = 0.717, Welch's *t*-test) (Table 3).

**Table 3:** Timing of inguinodynia detection and mean follow-up duration by surgical technique.

Technique	Inguinodynia	*N*	Detection time (months), Mean ± SD	Follow-up (months), Mean ± SD
TAPP	No	94	–	11.11 ± 2.67
	Yes	9	6.56 ± 3.43	9.00 ± 4.50
	Total	103	6.56 ± 3.43	10.92 ± 2.91
LICH	No	65	–	10.06 ± 3.50
	Yes	23	2.91 ± 0.85	12.65 ± 4.43
	Total	88	2.91 ± 0.85	10.74 ± 3.91
Overall	No	159	–	10.68 ± 3.07
	Yes	32	3.94 ± 2.51	11.63 ± 4.68
	Total	191	3.94 ± 2.51	10.84 ± 3.40
			*p* = 0.013*	*p* = 0.717**

TAPP, transabdominal preperitoneal repair; LICH, Lichtenstein tension-free hernioplasty.

* Welch's *t*-test for detection timing.

** Welch's *t*-test for follow-up duration.

### 3.4 Multivariate logistic regression analysis

Multivariate logistic regression analysis was conducted to identify independent predictors of inguinodynia, including variables such as diabetes, hypertension, BPH, smoking history, and surgical technique.

The analysis showed that undergoing LICH repair significantly increased the likelihood of developing inguinodynia compared to TAPP (adjusted OR = 2.98; 95% CI: 1.28–6.97; *p* = 0.012). Other variables, including smoking history, hypertension, diabetes, and BPH, did not independently predict inguinodynia. Due to low prevalence, asthma and COPD were excluded from the regression model to maintain reliability and statistical validity (Table 4).

**Table 4:** Multivariate logistic regression analysis for inguinodynia.

Variable	Coefficient (β)	SE	Wald χ^2^	*p*-value	Odds ratio (OR)	95% CI for OR
Intercept	−3.547	0.748	22.477	<0.001	0.029	–
Diabetes	−0.526	0.615	0.732	0.392	0.591	0.177–1.973
Hypertension	−0.330	0.536	0.379	0.538	0.719	0.251–2.056
Asthma^a^	–	–	–	–	–	Estimation not reliable^a^
COPD^a^	–	–	–	–	–	Estimation not reliable^a^
BPH	0.223	0.713	0.098	0.754	1.250	0.309–5.053
Smoking history	0.576	0.406	2.015	0.156	1.778	0.803–3.937
Type of surgery (LICH vs. TAPP)	1.093	0.433	6.374	0.012	2.984	1.277–6.974

LICH, Lichtenstein; TAPP, transabdominal preperitoneal; OR, odds ratio; CI, confidence interval; SE, standard error.

LICH was associated with higher odds of inguinodynia (OR = 2.98, 95% CI: 1.28–6.97, *p* = 0.012).

^a^ Estimates for asthma and COPD are unreliable due to very low case numbers.

## 4 Discussion

The present two-centre analysis highlights a consistent pain-sparing advantage for TAPP repair over LICH in routine elective practice. Although baseline age, sex and BMI were comparable, LICH patients carried a greater burden of hypertension and active smoking—co-morbidities already linked to impaired wound healing and heightened neural sensitivity (15, 16). Even after adjustment for these factors, LICH conferred an almost three-fold higher odds of chronic groin pain, reinforcing the operative approach itself as a dominant driver of inguinodynia in contemporary cohorts.

Emerging evidence indicates that laparoscopic hernia repair is associated with a markedly lower risk of chronic postoperative pain than open mesh techniques (17). Aasvang et al. reported persistent pain in 16% of 244 Lichtenstein repairs vs. 8.1% of 198 TAPP repairs (17), and a recent meta-analysis confirmed a significant long-term advantage for TAPP (*P* = 0.006) (18). The mechanistic basis appears to hinge on tissue trauma: the open approach routinely divides the cremasteric muscle and skeletonises the spermatic cord, whereas TAPP limits dissection to a controlled parietal-peritoneal flap. Corroborating this gradient, Bansal et al. noted more early pain after TAPP than after totally extraperitoneal (TEP) repair—attributing the discomfort to the additional peritoneal incision (19). In our series the mean onset of pain after LICH was three months, compatible with immediate nerve irritation; after TAPP symptoms surfaced later, mirroring scar-related traction. The ilio-inguinal branch was responsible for most neuralgia, as previously documented by Wright and Sanders (20). Earlier work also shows lower neuropathic intensity and faster resolution after laparoscopy (21–23), findings that dovetail with our numerical reductions.

Shorter hospitalisation after TAPP (mean 0.4 days) accords with randomised and observational studies demonstrating earlier mobilisation and return to activity following minimally invasive repair (11, 24, 25). Enhanced-recovery protocols that combine laparoscopy with regional blocks can compress in-patient stay to under 24 h in abdominal surgery (26); our experience suggests a similar trajectory is attainable for straightforward inguinal hernia repair. Although TAPP was applied more frequently for recurrent defects in the present study, registry data and matched comparisons indicate that, when mesh overlap is adequate, recurrence after TAPP approximates that of Lichtenstein repair (27–30). The primary technical pitfall after laparoscopy is insufficient medial coverage of the pubic tubercle (30, 31); fixation-free three-dimensional meshes may mitigate this risk while reducing foreign-body sensation (32).

Patient-related factors remain important modifiers. Smoking is independently associated with postoperative pain, recurrence and metachronous hernias (15), and tobacco exposure increases the incidence of general complications and prolongs hospital stay (16). Epidemiological studies further implicate age, body habitus and hiatal hernia in the genesis of inguinal defects and chronic pain (33). Genetic or developmental influences are suggested by the higher hernia prevalence documented in offspring of female survivors of childhood CNS tumours (34). These observations collectively argue for a personalised peri-operative strategy that combines risk-factor optimisation with meticulous operative technique.

Management of established inguinodynia is necessarily multimodal. Non-surgical measures predominate initially, but refractory cases may benefit from selective neurectomy or targeted mesh explantation, with laparoscopic TAPP neurectomy offering a safe and highly effective platform when conservative therapy fails (35–37). Recent reviews emphasise that both surgical factors (mesh position, fixation, nerve handling) and patient characteristics co-determine chronic pain trajectories, mandating thorough pre-operative counselling and expectation alignment (38).

In summary, our data add to a growing body of literature demonstrating that TAPP repair yields lower rates of chronic pain and shorter convalescence than Lichtenstein repair, without excess overall morbidity. Surgeons should therefore preferentially adopt laparo-endoscopic techniques when resources, expertise and patient profile permit, while maintaining strict attention to nerve preservation and mesh placement in all cases. Addressing modifiable risk factors such as smoking and hypertension may further attenuate the burden of inguinodynia and enhance functional recovery.

## 5 Limitations

This study has several inherent limitations due to its retrospective cohort design. Firstly, there is potential selection bias related to the choice of surgical technique, as surgeons might have selected TAPP preferentially for recurrent hernias or based on their expertise and experience. Secondly, the retrospective nature limited our ability to systematically assess postoperative pain using standardized pain scales, potentially influencing the accuracy and completeness of inguinodynia data. Thirdly, the follow-up duration, although a minimum of two years, may still be insufficient to capture long-term outcomes or late-onset complications comprehensively. Additionally, despite performing multivariate analyses to control for potential confounding factors such as diabetes, hypertension, and smoking, residual confounding from other unmeasured variables cannot be entirely ruled out. Lastly, the generalizability of our findings may be limited due to the relatively small sample size and the study's geographical restriction to two centers in Jordan, necessitating caution when extrapolating these results to broader populations.

## 6 Conclusion

Our study demonstrates that laparoscopic TAPP repair results in significantly less chronic inguinal pain and a modestly shorter hospital stay than open Lichtenstein hernioplasty, without increasing overall complications. Larger, multicentre trials with standardized pain metrics are needed.

## Data Availability

The raw data supporting the conclusions of this article will be made available by the authors, without undue reservation.
